# A neurocognitive approach to studying processes underlying parents’ gender socialization

**DOI:** 10.3389/fpsyg.2022.1054886

**Published:** 2023-01-09

**Authors:** Christel M. Portengen, Anneloes L. van Baar, Joyce J. Endendijk

**Affiliations:** Child and Adolescent Studies, Utrecht University, Utrecht, Netherlands

**Keywords:** gender socialization, parenting, gendered cognitions, neural processes, neurocognitive model

## Abstract

Parental gender socialization refers to ways in which parents teach their children social expectations associated with gender. Relatively little is known about the mechanisms underlying gender socialization. An overview of cognitive and neural processes underlying parental gender socialization is provided. Regarding cognitive processes, evidence exists that parents’ implicit and explicit gender stereotypes, attitudes, and gendered attributions are implicated in gender socialization. Other cognitive factors, such as intergroup attitudes, gender essentialism, internal motivation for parenting without gender stereotypes, gender identity, and conflict resolution are theoretically relevant mechanisms underlying gender socialization, but need further investigation. Regarding neural processes, studies demonstrated that attentional processing, conflict monitoring, behavior regulation, and reward processing might underlie stereotypes and biased behavior. However, more research is necessary to test whether these neural processes are also related to parental gender socialization. Based on this overview, a framework is presented of neural and cognitive factors that were theoretically or empirically related to gender socialization.

## Introduction

Gender is an important category that shapes children’s social lives (Blakemore et al., 2008). This starts already before birth, when parents decorate the baby’s room, or decide upon the name the baby is given. These decisions represent the first indications of parental gender socialization, which comprises all intentional and unintentional ways in which parents teach their children the social expectations and attitudes associated with gender (Henslin, 1981; Endendijk et al., 2018). Parents can employ several types of gender socialization. First, parents can (unintentionally) create gender-specific environments for children through the provision of activities, chores, books, toys, resources, or opportunities (i.e., channeling or shaping; Blakemore et al., 2008; Dittman et al., 2022). Second, parents may use different parenting practices with their sons and daughters, which is known as gender-differentiated parenting (Endendijk et al., 2016). Third, parents appear to respond more negatively to behavior that violates gendered expectations (e.g., a boy who plays with dolls) than when gender stereotypes are confirmed (e.g., a boy who plays with cars; Smetana, 1989; Morrongiello and Dawber, 2000; Martin and Ross, 2005). Fourth, parents serve as models for appropriate gender-role behavior through their own behaviors, interests, and division of work and household tasks (Bandura, 1969; Bandura and Walters, 1977; Bussey and Bandura, 1984, 1999; Endendijk and Portengen, 2021). Fifth, parents may use gendered communication, such as gender labeling (e.g., boy, girl, he, she) or evaluative comments that emphasize the appropriateness of gender-typical behaviors (e.g., “Look, those girls are fighting. That is not nice!”) (Endendijk et al., 2014). Importantly, it was argued by Mesman and Groeneveld (2018) that “gender socialization is expressed primarily in specific parenting practices (rather than broad parenting styles) and mostly implicitly (rather than explicitly)” (Mesman and Groeneveld, 2018, p. 23).

There is ample evidence that parental gender socialization is associated with the development of gender stereotypes (Halpern and Perry-Jenkins, 2016), as well as gender differences in language skills (Pruden and Levine, 2017), academic achievements (Updegraff et al., 1996), occupational preferences (Sandberg et al., 1991), and problem behaviors (Endendijk et al., 2017) in children and adolescents. Even though there is a large body of research demonstrating the consequences of parental gender socialization for the (gender) development of children and adolescents (for a review, see Endendijk et al., 2018; Morawska, 2020), we still know relatively little about the factors and mechanisms underlying and explaining gender socialization. However, more insight into these underlying mechanisms would lead to a better understanding *why* some parents are more likely to employ gender socialization with their children than others. Moreover, these mechanisms can be targeted in parenting interventions aimed at reducing gender inequality in future generations of children.

Neurocognitive frameworks and research could provide valuable insights into the underlying mechanisms of gender socialization for several reasons. First, parental gender socialization has been characterized as a rather implicit process (Mesman and Groeneveld, 2018). ‘Implicit’ in this context indicates that parents might not be aware that they convey gendered information to their children, that parents might not have the intention to transmit gendered information, or that gender socialization is expressed in a relatively automatic way (Gawronski et al., 2009). Neurocognitive measures might be better able to capture such subconscious processes than self-report or behavioral measures (Greenwald et al., 2002). In addition, a neuroscientific approach is recommended when examining the intuitive/automatic processes underlying parenting (Parke, 2017). More specifically, neuroscientific research can provide insights in the temporal dynamics underlying parenting as well as the brain areas and processes involved in parenting (Maupin et al., 2015).

Neuroscience might not only add to the understanding of gender socialization, but neurocognitive research on gender socialization could also inform neuroscience, by building a bridge between neuroscientific measures and actual parenting behavior. This could improve the ecological validity of neuroscience (Derks et al., 2013; Feldman, 2015). In addition, neuroscientific research on gender stereotyping has focused primarily on people’s responses to unfamiliar adult men and women. It is not yet known whether the same neural processes are also involved when people respond to their own sons and daughters with whom they have a strong emotional connection. Neuroscientific research on gender socialization could answer such questions.

Therefore, this paper reviews what is known about cognitive and neural processes underlying parental gender socialization of children and adolescents, and how these processes can be measured. The goal of this narrative review is not to provide an exhaustive overview of existing research on this topic. Instead, we aim to guide and inspire future research and theory building on the neurocognition of gender socialization, by describing multiple relevant neural and cognitive processes that might be implicated. For some of these processes evidence is already found, but others seem theoretically relevant to study in relation to gender socialization. Throughout this paper the term gender is used to reflect the social meaning attached to a person’s biological sex. As the vast majority of research on neurocognitive processes underlying gender socialization takes a binary approach, contrasting males and females, this gender binary is also reflected in the current review. Greater representation of the unique experiences of transgender and nonbinary parents and children remains an important direction for future research on gender socialization.

In this review, we first build on gender schema theories (GSTs; Bem, 1981; Martin and Halverson, 1981; Bem, 1983) and neural models of stereotypes (Amodio, 2014) to identify several neural and cognitive processes that may explain why some parents are more likely to apply gender socialization practices than other parents. Subsequently, empirical evidence for direct associations between cognitive and neural processes and gender socialization is discussed. As this body of literature is small, we will thereafter describe empirical evidence for cognitive and neural processes associated with gendered behavior in general, as these processes might also be implicated in parental gender socialization. We conclude with a summary of the available evidence and directions for future research.

### Theoretical underpinnings of cognitive and neural processes in gender socialization

Two theoretical frameworks provide predictions about the neurocognitive processes that might be associated gender socialization, namely gender schema theories and neural models of gender stereotypes.

#### Gender schema theories

First, from GSTs (Bem, 1981; Martin and Halverson, 1981; Bem, 1983) it can be argued that several cognitive processes might play a role in parents’ gender socialization. Gender schemas are cognitive structures containing gender-related information that shape one’s processing of the social environment. Although GSTs primarily focus on the link between gender cognitions and gendered behavior and experiences in children, the basic principles can also be applied when trying to explain the mechanisms behind parental gender socialization.

The most relevant prediction from GSTs for explaining parental gender socialization is the idea that gender schemas provide cognitive social standards that guide behavior. Applied to parental gender socialization, this means that parents might use gender socialization to align their children’s preferences and behaviors with the culturally determined gender norms or their own gender cognitions. However, there are individual differences in the strength or traditionality of people’s gender cognitions (Bem, 1981, 1983). In particular, strong gender cognitions may lead to parental gender socialization that suppresses the child’s own interests, skills, and behaviors that do not conform to parents’ gender schemas (Bem, 1981, 1983; e.g., suppress doll-play in boys but not in girls). Yet, parents with less strong gender cognitions about boys and girls might be more likely to show egalitarian socialization of their children (e.g., do not treat boys and girls differently, emphasize similarities between boys and girls). GSTs also posit that once gender cognitions become a prescriptive guide, an internalized motivation prompts a person to regulate their behavior (Bem, 1981). This internal motivation encourages a person to regulate their behavior so that it conforms to their gender schemas. In the context of gender socialization, this internalized motivation may entail a parents’ motivation for parenting without gender stereotypes.

Several types of interrelated gender cognitions exist that all concern the way people think about themselves and others in terms of gender (Bugental and Johnston, 2000; Tenenbaum and Leaper, 2002). Because gender cognitions are multi-dimensional, this review summarizes evidence for a broad range of gender cognitions. We focus on the following most studied gender cognitions: parents’ gender stereotypes and gender attitudes, gendered attributions, gender essentialism, gender identity, internal motivation for parenting without stereotypes, and conflict resolution.

#### Neural model of implicit stereotypes

In addition, neuroscientists have developed a neural model of implicit stereotypes (Stanley et al., 2008; Amodio, 2014) reflecting several neural processes that could underlie parental gender socialization. In this neural model, the *temporal pole* functions as a hub for social (stereotype) knowledge (Olson et al., 2013). Based on this stereotype knowledge, the *amygdala* automatically evaluates socially salient (both negative and positive) stimuli and facilitates the allocation of the appropriate attentional processes to respond (Amodio, 2014). However, relying solely on automatic evaluations to drive our behaviors is not an optimal strategy in our complex social environment, and a certain level of control over the influence of stereotypes on behavior would be necessary. Therefore, the *anterior cingulate cortex (ACC)* is thought to monitor conflict between the automatic evaluations of a stimulus with the person’s expectations of that stimulus. For instance, when a parent expects boys to be tough but encounters a crying boy, conflict arises, which is signaled by the ACC. When conflict arises, the ACC in turn activates the dorsolateral *prefrontal cortex (dlPFC) and dorsomedial prefrontal cortex (dmPFC)* to resolve the conflict (Stanley et al., 2008; Cattaneo et al., 2011). These prefrontal brain structures, together with the striatum and motor cortex, then regulate a person’s behavioral responses, allowing one to overcome the expression of gender stereotypes (Cattaneo et al., 2011).

In the context of gender socialization, the amygdala’s role in signaling salience may be particularly relevant (Santos et al., 2011). The amygdala might become activated in response to a son or daughter violating gender expectations, as such stereotype violations are salient. Increased salience processing of unexpected behavior might explain parents’ negative responses to children’s behavior that violates gender expectations (e.g., Sandnabba and Ahlberg, 1999; Endendijk et al., 2014). However, when top-down (ACC and dmPFC/dlPFC) conflict-monitoring and behavior regulatory mechanisms are activated, parents might be able to overcome their first automatic response and inhibit negative responses to boys’ and girls’ gender-atypical behavior (Li et al., 2016).

Thus, neural models of gender stereotypes point to the following processes as possibly underlying parental gender socialization: parents’ gender knowledge (i.e., type of gender cognition), attention allocation processes, conflict resolution mechanisms, and behavioral regulation mechanisms.

## Materials and methods

A narrative review was conducted to provide an overview of the available information on cognitive and neural processes that may be underlying gender socialization. A narrative review is different from a systematic review in that it is not aimed to be systematic or exhaustive, but instead provides an overview of the state-of-the-art in a certain field of research. The goal is to guide future theory building and research in the field. As recommended by Lilford et al. (2001), a wide range of databases and sources were used for our literature search. Second, Lilford et al. (2001) have recommended to allow overlap in the stages of the review process, while differentiating the phases of searching, analyzing, and writing up of the review report. This recommendation allows the researchers to refine concepts concerning the nature and scope of the review. These principles were applied in our search strategies for articles to be included in this narrative review.

The following process was used for the literature search. First, terms were identified on the basis of two relevant theoretical models (i.e., the GSTs and the neural model of implicit stereotypes), as well as the authors’ expert knowledge of literature on gender socialization. For cognitive processes, search terms included: gender cognitions, (parents) gender stereotypes, (parent) gender attitudes, gendered attributions, gender essentialism, gender identity, and internal motivation to respond without prejudice. For the neural processes, we used neuroscientific measurement terms (electroencephalography, functional MRI, TMS) combined with (gender) stereotypes, (gendered) parenting, or gender socialization. These terms were entered in Google Scholar, Scopus, and Web of Science to search for literature regarding these terms in relation to gender socialization. Moreover, we have used the citation and reference lists of relevant articles to identify research that could be related to our topic. In a second stage, other terms were added to the literature search. For cognitive processes, these terms included intergroup relations, conflict resolution, and (benevolent) sexism. For neural processes, search strategies were broadened to include racial stereotypes and attitudes, as well as the relation between neural processes and parenting in general. This was done to obtain a more comprehensive image of neural processes, since the neuroscientific literature on gender socialization is scarce. The first and last authors together decided on the inclusion and exclusion of articles in the review. The main inclusion criterium was that a type of cognitive or neural process was examined and related to gender socialization, gendered behavior, or (gender) stereotyping.

## Empirical evidence for cognitive processes implicated in parental gender socialization

For several cognitive processes proposed by GST’s as underlying parents gender socialization direct empirical evidence has been found. This will be discussed separately for the different cognitive processes.

### Parental gender stereotypes and attitudes

A stereotype is “the association of a social group with one or more (non-valence) attribute concepts” (Greenwald et al., 2002). Applied to gender, the social categories are men/boys and women/girls, and attribute concepts often relate to the behaviors, roles and characteristics that are typically associated with men or women. A gender attitude refers to people’s positive and negative evaluations of the behaviors, roles and characteristics for men and women (Greenwald et al., 2002). Gender stereotypes and attitudes can be present at both an explicit and an implicit level (Gawronski and Creighton, 2013). Explicit stereotypes and attitudes are overtly expressed ideas that are under conscious control and, therefore, are especially prone to social-desirable responding (Greenwald et al., 2009). Implicit stereotypes and attitudes, on the other hand, are supposedly relatively inaccessible to conscious awareness, are elicited unintentionally, require few cognitive resources, and cannot be stopped voluntarily (Gawronski and Bodenhausen, 2006). Implicit stereotypes and attitudes are therefore most often assessed with response latency measures. For such measures is assumed that performing congruent tasks in which responses and stereotypes/attitudes are aligned require less effort and can be performed faster, compared to incongruent tasks reflecting stereotypes/attitudes and responses that do not align.

A widely used response latency measure to assess implicit gender stereotypes and attitudes is the Implicit Association Test (IAT; Rudman et al., 1999; Greenwald and Krieger, 2006). IATs measure the strength of (automatic) cultural associations between concepts (e.g., boys, girls, men, women) and attributes (e.g., male-typed toys, female-typed toys, science, career, family). The validity of the IAT is, although criticized, well-documented (Bluemke and Friese, 2008; Greenwald et al., 2009).

In a study that measured parents’ gender stereotypes about career and family with an IAT, fathers with stereotypical IAT scores (i.e., associating career with men and family with women) used more physical control strategies with their 3-year-old sons than with their 3-year-old daughters (Endendijk et al., 2017). On the other hand, fathers with counter-stereotypical IAT scores (i.e., associating career with women and family with men) used more physical control strategies with daughters than with sons (Endendijk et al., 2017). Individual differences in parents’ implicit gender stereotypes might thus be related to individual differences in gender-differentiated parenting.

In another study, parents’ gender stereotypes about toys were assessed with a task similar to the IAT and gender socialization was captured during picture book reading (Endendijk et al., 2014). Mothers with stronger implicit gender stereotypes were more likely than mothers with more egalitarian stereotypes to employ gendered communication that emphasized gender stereotypes toward their preschool children. More specifically, they made more comments confirming gender stereotypes, they evaluated gender-role inconsistent behavior more negatively, and they used gender labels to convey the stereotype-congruent nature of the activities in the pictures (e.g., using the masculine label for gender-neutral children playing with water guns). Together, these studies provide evidence for the idea that implicit gender stereotypes are a mechanism underlying parents’ gender socialization practices.

Even though implicit cognitions are often better predictors of behavior than explicit cognitions (Greenwald et al., 2009), there are several studies that find associations between explicit gender stereotypes or attitudes and parents’ gender socialization as well. These studies provide further support for gender stereotypes and attitudes being an important mechanism underlying gender socialization of children and adolescents. For instance, stronger gender stereotypes about toys were associated with less nontraditional toy purchases in prospective parents (Weisgram and Bruun, 2018). Also, mothers who reported having egalitarian gender-role attitudes made more counterstereotypical comments during book reading (e.g., “Girls can also build igloos!”) toward their preschool children than mothers who reported more traditional gender-role attitudes (Friedman et al., 2007). In addition, parents with egalitarian gender-role attitudes found cross-gender-typed toys more desirable for their preschool children than did parents with traditional gender-role attitudes (Kollmayer et al., 2018).

In middle childhood, more traditional gender attitudes were associated with a more gender-stereotyped division of labor between parents (i.e., modeling aspect of gender socialization; McHale et al., 1999) as well as with encouragement of gender-typed behaviors in their children (Raffaelli and Ontai, 2004), but children’s felt pressure from parents to conform to gender roles appeared unrelated to parents’ gender socialization attitudes (Schroeder and Liben, 2021). Also in middle childhood, parents with stronger math-gender stereotypes provided more intrusive support to middle school girls during math homework (Bhanot and Jovanovic, 2005) and were involved in their daughter’s math homework (Denner et al., 2016). In adolescence, more traditional gender-role attitudes in mothers were associated with more conservative child rearing practices that taught daughters to comply with traditional norms and values (Ex and Janssens, 1998), as well as with granting girls fewer autonomy opportunities than boys (Bumpus et al., 2001). However, mothers with more traditional gendered beliefs were not found to differentiate between boys and girls.

### Parents’ gender attributions

Next, to gender stereotypes and attitudes, parents may hold different attributions of the intentions, behaviors, gendered goals, and appropriateness of responses of their sons and daughters (Endendijk et al., 2018; Bugental and Corpuz, 2019). Gendered attributions are the gender-differentiated inferences and beliefs parents have about the causes of their children’s behaviors, achievements, and preferences. Gender attributions differ from gender stereotypes in that they concern the roots of people’s achievements and behaviors, rather than the preferences and behaviors itself (Reyna, 2000). Parents’ attributions of the behavior of boys and girls can be measured with vignettes, scenarios, or pictures showing boys and girls in different behaviors (Morrongiello and Rennie, 1998; Morrongiello and Hogg, 2004). In a study using a scenarios of risk behavior, parents of preschoolers believed that boys’ risky behaviors are inborn, whereas girls’ risky behaviors were triggered by situational factors (Morrongiello and Rennie, 1998; Morrongiello et al., 2010). Consistent with these attributions, parents believed that daughters can be taught to comply with safety rules more than sons (Morrongiello et al., 2010), and parents would supervise and actively try to prevent risky misbehavior to daughters, but not to sons in middle childhood (Morrongiello and Hogg, 2004; Morrongiello et al., 2008). Apparently, mothers’ gendered attributions about the fixed/malleable nature of boys’ or girls’ characteristics might explain whether mothers used gender-differentiated parenting practices to prevent risky behavior.

## Evidence for cognitive processes that underlie gendered behavior in general

Previous research has established that several types of gender cognitions, such as gender stereotypes and attitudes and parents’ gendered attributions were associated with parents’ gender-differentiated parenting. It seems plausible that other cognitive processes might also play a role in parental gender socialization. These cognitive processes are, however, hardly studied in the context of gender socialization.

### Gender identity

Parents’ own gender identity could also play a role in their gender socialization practices. Gender identity refers to one’s sense of being male or female and provides an important basis for people’s interaction with others (Steensma et al., 2013), and is most often assessed via self-report (e.g., Dinella et al., 2014). In general, gender identity is thought to foster behavior in line with gender roles (Taylor and Hall, 1982). Yet, gender identity might also explain variability in behavior because gender identity differs across individuals (Wood and Eagly, 2015). Applied to gender socialization this could mean that parents who strongly identify with their own gender might socialize their children into traditional gender roles. In adults, gender identity has been associated with several gender-typed behaviors and cognitions (Wood and Eagly, 2015). For instance, feminine gender identity has been associated with greater involvement with family roles (Abele, 2003). In addition, self-perceived gender typicality (one of the dimensions of gender identity) was related to more gender-typical career interests in both men and women (Dinella et al., 2014). It is yet unclear whether gender identity is also associated with other forms of parental gender socialization.

### Intergroup attitudes

Intergroup attitudes can be defined as the tendency to evaluate one’s own membership group (the in-group) more favorably than a non-membership group (the out-group) (Tajfel and Turner, 1986). Intergroup attitudes can be measured with self-report questionnaires assessing people’s evaluation of the in-group and out-group, or with Implicit Association Tests in which participants have to pair positive and negative attributes to the ingroup and outgroup (Greenwald and Pettigrew, 2014). We know that adults implicitly and explicitly evaluate their own gender positively and the other gender more negatively (Rudman and Goodwin, 2004; Dunham et al., 2016), which is associated with discriminative behavior to outgroup members (for a review, see Greenwald and Pettigrew, 2014). However, it is not known whether parents’ in-group favoritism also transfers to different treatment of same-gender offspring compared to opposite-gender offspring. There is some evidence in the preschool period that mothers who endorsed hostile sexist attitudes, which might be related to in-group favoritism, had stronger maternal gatekeeping tendencies, which resulted in a greater maternal share of childcare tasks relative to the father (i.e., modeling aspect of gender socialization; Gaunt and Pinho, 2018).

### Gender essentialism

Gender essentialism is the idea that “members of a category share an inherent, non-obvious property (essence) that confers identity and causes other category-typical properties to emerge” (Gelman et al., 2004). People with essentialist beliefs consider gender differences to be innate (rather than environmentally evoked) and thus fixed (instead of malleable), and are often more inclined to support gender discriminatory processes and endorse gender inequalities (Skewes et al., 2018). Essentialists beliefs are predictive of gender stereotype endorsement in both non-parents (Bastian and Haslam, 2006) and parents (Meyer and Gelman, 2016). Of interest to the current review was that parents’ gender essentialism was associated with young children’s gender-typed preferences (Meyer and Gelman, 2016). Parental gender socialization might mediate this association, such that parents with strong essentialist beliefs may reinforce or shape children’s behaviors toward more gender-typical preferences (Meyer and Gelman, 2016). However, it is also possible that having children with strong gender-typed preferences might fuel parents’ gender essentialist thinking. Essentialist thinking has been associated with a more traditional division of household tasks between parents in families with preschool children (Pinho and Gaunt, 2021). Longitudinal research, examining direct relations between parents’ gender essentialism and gender socialization while controlling for children’s gender-typed behavior, is necessary to determine whether gender essentialism indeed underlies parental gender socialization.

### Conflict resolution

Another relevant cognitive process is conflict resolution. The idea is that when people have to categorize clear, or stereotype-congruent, exemplars of a category (e.g., a masculine boy) they experience less internal conflict than when they have to categorize less clear, or stereotype-incongruent, exemplars of a category (e.g., a feminine boy). Parents might experience conflict when their child shows behavior that is not in line with the stereotyped expectancies they have about the appropriate behavior of boys and girls (Endendijk et al., 2019b). When they are unable to resolve this internal conflict, they might use gender socialization practices aimed at aligning the behavior of their child with their stereotyped expectancies, and thus restore conflict.

Conflict resolution can be captured with the use of mouse-tracking paradigms. In general, mouse-tracking paradigms require people to categorize (visual) stimuli onto two categories presented in the left and right corners of a screen. The trajectory they make with the mouse when dragging a stimulus to one of the categories is captured. When the trajectory deviates from a straight line between the stimulus and the category this provides indications of response conflict, as well as whether decisions are made relatively automatically and then consciously confirmed or overridden (Stillman et al., 2018).

Mouse-tracking has not been used yet to explain parents’ gender socialization practices. But there is some evidence that mouse-tracking trajectories indeed are associated with actual gendered behavior in non-parents (Hehman et al., 2014a). Hehman et al. (2014a) examined whether gendered facial attributes of U.S. female politicians were associated with the likelihood of being voted for during elections. They found that when female politicians’ faces were more gender-incongruent, participants experienced more conflict assigning the face to the female category, as evidenced by a larger slope in the observed mouse trajectory. In addition, participants were less likely to vote for these female politicians, but this was not the case for male politicians. Moreover, this effect was even more pronounced in more conservative areas in the U.S. (Hehman et al., 2014a).

### Motivation for parenting without gender stereotypes

Parents’ motivation for parenting without gender stereotypes forms another relevant factor to study in relation to gender socialization. One’s motivation to respond without prejudice or bias is theorized to function as a buffer for expressing stereotypes or behaving in accordance with stereotypes (Plant and Devine, 1998). This motivation can be both internal and external. External motivation depends on social pressure to inhibit the overt expression of stereotypes. Internal motivation represents underlying, intrinsic motivations to respond without prejudice irrespective of the situational pressures. It might be most relevant to relate parents’ internal motivation to their implicit gender socialization practices since gender socialization frequently takes place when parents are at home with their children. In this context social pressures are unlikely to play a role. Parents with higher internal motivation for parenting without gender stereotypes might be less likely to use gender socialization that steers boys and girls into traditional gender roles (Plant and Devine, 1998).

Evidence exists that internal motivation to respond without stereotypes contributes to less stereotyped behavior in two ways (Amodio and Swencionis, 2018). First, internal motivation can suppress the activation of stereotypes, for instance when a parent’s son wants to play with dolls. This process is found to be preconscious and might prevent the activation of the stereotype ‘boys do not play with dolls’ (Amodio et al., 2008) and subsequently prevent a parent’s negative response to the gender-atypical behavior of their son. However, it might not always be possible to completely avoid the activation of gender stereotypes because of external influences (e.g., children making stereotyped comments) or internal influences (e.g., cognitive overload; Amodio and Swencionis, 2018). Once stereotypes do get activated, internal motivation can also support the intentional control of gender stereotypes over behavior. In the context of gender socialization this could mean that when parents hold stereotyped expectancies about the behavior of boys and girls, these stereotypes could get activated by the behavior of their sons and daughters. However, when parents have a strong internal motivation for parenting without gender stereotypes this motivation might suppress the influence of gender stereotypes on their parenting behavior.

Although there is ample evidence that internal motivation to respond without prejudice is related to less stereotyped behavior in interracial relations (Butz and Plant, 2009), this has not been examined in the gender socialization context. In order to study this factor in a gender socialization context some adaptation might be needed, for instance by conceptualizing it as parents’ motivation for parenting without gender stereotypes. A recent study in parents found that mothers’ internal motivation to behave without gender stereotypes appeared unrelated to how mothers’ evaluated preschool boys’ and girls’ stereotypical and counter-stereotypical toy play (Endendijk et al., 2019a). However, both the internal motivation measure as well as the toy-play evaluation measure concerned boys and girls in general, and not mothers’ own sons and daughters (Endendijk et al., 2019a). It may be more relevant to measure if parents’ internal motivations for parenting without gender stereotypes is related to gender socialization practices with their sons and daughters.

### Domain-specificity of gender cognitions

Studies linking parental gender cognitions to gender socialization practices thus far have focused primarily on parents’ stereotyped expectancies and attitudes about boys’ and girls’ toy and activity preferences and academic abilities. However, gender cognitions can span multiple domains, which might be specifically linked to different types of gender socialization. For example, parents gender stereotypes about toys and activities might be specifically related to the toys that parents provide their children with and the activities they involve their children in. However, adults also hold different explicit expectations about children’s personality traits and behaviors (Martin, 1995). For instance, they rate some emotions and behaviors, such as crying, being easily frightened, to be less desirable for boys, and other behaviors, such as being noisy, as less desirable for girls (Martin, 1995). These expectations about the appropriateness of certain *emotions and behaviors* for boys and girls might specifically explain whether parents socialize girls and boys to show different *emotions* (Fivush et al., 2000; Chaplin et al., 2005; van der Pol et al., 2015) or to exhibit different *behaviors* (Endendijk et al., 2017). Together, these studies highlight the importance of examining associations between parents’ gender cognitions and gender socialization practices in a domain-specific way.

## Empirical evidence for neural processes associated with parental gender socialization

Researchers have used both functional magnetic resonance imaging (fMRI) and electroencephalography (EEG) to identify the neural correlates of gender socialization. Each measure has its own advantages. Functional imaging studies have the benefits of a high spatial resolution, meaning that they are better at localizing activity in certain brain areas. EEG, on the other hand, provides a high temporal resolution, which enables researchers to capture the implicit nature and temporal dynamics of parenting (Maupin et al., 2015). Summarizing the findings of both methods will provide a more complete and detailed image of neural processes underlying parental gender socialization. There are only a handful studies that assessed the neural processing of gendered stimuli, and even fewer studies who examined this in parents. Therefore, we also present evidence in non-parents for the neural networks and processes associated with stereotypes and stereotyped responses in general in the next section.

People’s neural responses to stimuli that violated social expectations have generally been studied using three paradigms. First, several studies have used Implicit Association Tests (e.g., Healy et al., 2015). These studies examined whether neural responses differed between trials in which words/pictures had to be categorized in a way that was consistent with social expectations and trials in which words/pictures had to be categorized in a way that violated social expectations. Second, other studies used passive viewing paradigms (e.g., Endendijk et al., 2019a). In such paradigms, participants were asked to look and form impressions of pictures showing people violating social expectations or people confirming social expectations. Differences in brain activity between the two types of pictures were examined. Third, studies have used priming paradigms (e.g., Hehman et al., 2014c). For example, participants were shown pictures of men or women that were primed with words that either violated or confirmed social expectations. Participants had to categorize the pictures as male or female. Brain activity was compared between the trials that violated versus confirmed social expectations. These tasks are similar to the tasks used to assess parents’ gender stereotypes and attitudes that were discussed in the section on cognitive processes.

Studies using EEG to examine neural correlates of gender stereotypes and stereotyped behavior are often designed to capture event-related potentials (ERPs). ERPs are epochs of neural activity that are time-locked to the presentation of a stimulus and measured by electrodes. It is somewhat speculative to which neural processes ERPs refer, but studies over the years have associated such event-related activity to several functions in the brain.

The one study that specifically related ERPs elicited by gender-congruent versus incongruent stimuli to mothers’ gender communication with their own children found evidence for the importance of early attentional processing in gender socialization (Endendijk et al., 2019b). Differences in P300 and N2 activity between gender-congruent (e.g., associating a toy car with a boy) and incongruent (e.g., associating a doll with a boy) stimuli were found to be related to the mothers’ gendered communication with their preschool children (Endendijk et al., 2019b). N2 activity reflects overcoming stereotypical responses (i.e., *conflict resolution*) or *conflict monitoring* (Azizian et al., 2006). The P300 is thought to reflect processes such as *response selection* under difficult conditions (Twomey et al., 2015) and *attention allocation* to stimuli that are negatively valenced, surprising, or unexpected (Bartholow and Dickter, 2007; Polich, 2007). In addition, these differences in early neural processing were more robustly related to gendered communication than their level of implicit or explicit gender stereotypes (Endendijk et al., 2019b). Together, these findings demonstrated that gendered communication is indeed an unconscious process. In addition, parents’ attention allocation to gendered stimuli, more specifically attention to unexpected gender stimuli and attention to gender stimuli that parents evaluated as positive, might underlie gendered communication.

One functional magnetic resonance imaging (fMRI) study in fathers also provides evidence for the assumption that neural responses to gender stimuli are associated with real-world parenting behaviors. In this study, fathers of daughters were more attentively engaged, sang more, and used more analytical language and language related to sadness and the body with their daughters, than fathers of sons (Mascaro et al., 2017). In contrast, fathers of sons spend more time in rough and tumble play (RTP) and used more achievement language with their sons than did fathers of daughters. Additionally, fathers of daughters showed elevated medial and lateral OFC (mOFC and lOFC) responses toward their daughters’ happy facial expression, whereas fathers of sons showed elevated mOFC responsivity toward their sons’ neutral facial expressions. More importantly, mOFC activity in response to happy facial expressions was negatively associated with the amount of time fathers engaged in RTP, whereas the mOFC responsivity toward neutral faces was positively associated with more time spend in RTP for fathers of sons specifically (Mascaro et al., 2017). The mOFC has been implicated in reward processing (Rolls et al., 2020). Hence, parents’ *reward processing* of the emotional faces of their sons and daughters might underlie differences in play styles with their sons and daughters.

## Evidence for neural processes underlying (gender) stereotyping in general

### EEG research

Research on people’s temporal processes toward the violation of social expectations have pointed toward several other ERPs than the previously mentioned N2 and P3 that might be relevant in the context of gender socialization. The first are *early attentional processes* reflected by peak P100, N170, and P200 amplitude. The P100, N170 and P200 ERPs were found to be elicited by out-group faces during an IAT (He et al., 2009) and by behaviors violating expectations during an impression formation task (Dickter and Gyurovski, 2012; Rodríguez-Gómez et al., 2020). Second, the late positive potential (LPP) which reflects *attentional orienting* to salient stimuli (Huffmeijer et al., 2014).

EEG studies on the neural correlates of gender stereotypes in (non-)parents have found several indications of altered early-stage processing in occipital and frontal lobes that were associated with different types of (gender) cognitions. For example, Healy et al. (2015) found larger N2 amplitudes during congruent trials than incongruent trials, specifically in people with medium stereotype scores. Regarding the P200, people with stronger racial biases demonstrated greater P200 activity to incongruent racial stimuli (e.g., black face primed with white trait) than to congruent racial stimuli (e.g., black face primed with black trait; Hehman et al., 2014c). Regarding the LPP, differences in LPP activity to gender-stereotype congruent and incongruent sentences were associated with adults’ hostile sexism (Canal et al., 2015). However, differences in N170 and LPP to gender congruent and incongruent sentence-face combinations were found to be unrelated to adults’ level of sexism (Rodríguez-Gómez et al., 2020). Together these studies indicate that *early attentional processing* of stimuli that confirm of violate stereotyped expectations and salience processing might underlie stereotypes and stereotyped behavior in general.

Although limited, there are some studies that have implicated brain activity epochs and activation patterns with actual behaviors. For example, one study associated N2 amplitude differences in fronto-central areas during a prosocial attitude IAT with actual donating behaviors (Xiao et al., 2015). The researchers found that people who showed increased N2 activity in response to incongruent trials (associating prosocial words with “others” and non-prosocial words with “self”) on the prosocial IAT, donated more than people who showed increased N2 activity in response to congruent trials (associating prosocial words with “self” and non-prosocial words with “others”). The increased N2 activity found in this study might reflect increased *attention* to stimuli that fit with peoples’ prosocial (or self-oriented) behavioral tendencies.

### fMRI research

Research on the neural activation patterns of adults when they had to categorize stimuli that confirm or violate stereotypical expectations have shown elevated neural activation in behavioral regulation networks (Knutson et al., 2007; Mitchell, 2008; Quadflieg et al., 2011). For instance, when non-parents categorized targets that were inconsistent with their gender-stereotypes, the *dmPFC*, middle temporal gyrus and the posterior cingulate cortex showed enhanced activation (Quadflieg et al., 2011). *Medial PFC and ACC* regions were also activated while non-parents had to categorize stereotype-congruent gender and race stimuli, whereas the *dlPFC* was recruited when participants were asked to categorize stimuli that were incongruent with their stereotypes (Knutson et al., 2007). Importantly, activation of the *dlPFC* in response to stereotype violating stimuli was associated with the strength of people’s stereotypes (Hehman et al., 2014b). Activation of the *dmPFC* cortex was found in response to stereotype violating racial stimuli, but a stronger internal motivation to respond without prejudice attenuated the dmPFC response (Li et al., 2016). In addition, enhanced *amygdala* activation was found during gender-congruent trials (Knutson et al., 2007). Activity in the *anterior temporal lobe* (*ATL*; part of the temporal pole) has also been associated with both implicit racial stereotypes and attitudes assessed with IATs (Gilbert et al., 2012). However, it is unclear whether the ATL might also play a role in both the evaluative component (i.e., attitudes) and the associative component (i.e., stereotyping) of parents’ gender cognitions (Gilbert et al., 2012). The temporal pole is presumed to be critical for *linking person-specific memories to faces* (Olson et al., 2013) and might therefore also play a role in the memories of gender-typical and atypical behavior that parents link to their child’s face.

When examining the neural processing of gender stereotypes in mothers of young children, both the *dmPFC and the ACC* have shown larger BOLD changes pictures of children combined with stereotype-incongruent toy words (Endendijk et al., 2019a). The elevated ACC activity was also associated with stronger gender stereotypes in mothers, most likely reflecting the ACC’s role in conflict monitoring. Additionally, in mothers, the left temporoparietal junction (TPJ) responded specifically when incongruent toy words were paired with boy faces (Endendijk et al., 2019a). The larger TPJ activation may reflect the more restrictive gender norms for boys (Sandnabba and Ahlberg, 1999; Kane, 2006), since the TPJ is often activated when social expectations are violated (Cloutier et al., 2011). These results indicate that mothers might *experience conflict* when a child’s behavior does not match their gender stereotypical expectations, but how this transfers to actual gender socialization practices with their own children is largely unknown.

## Summary of findings and future directions

**Figure 1:**
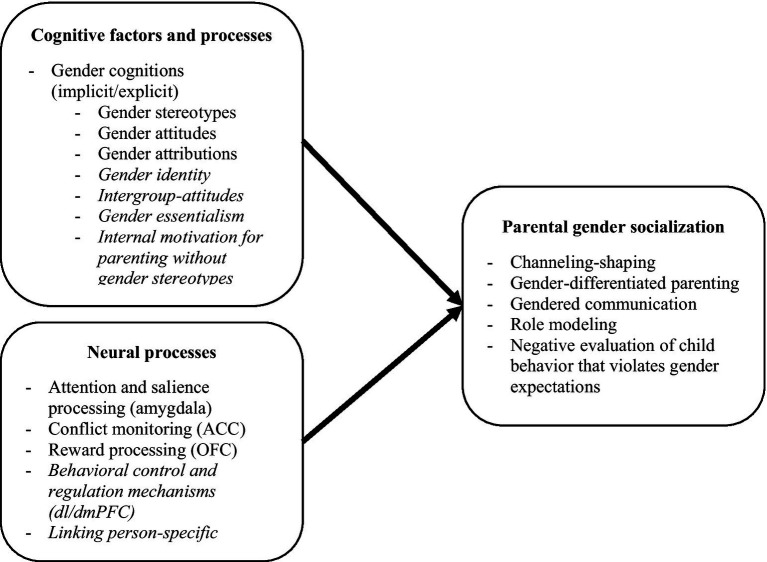
Overview of neural and cognitive processes underlying parental gender socialization. Cognitive and neural processes written in italics are processes for which there is only theoretical support and/or indirect empirical evidence linking these processes to other types of stereotyped behavior than gender socialization. For processes and factor that are not in italics, there is direct evidence of a link with parental gender socialization. The following abbreviations are used in the model: anterior cingulate cortex (ACC), dorsolateral and dorsomedial prefrontal cortex (dl/dmPFC), orbitofrontal cortex (OFC), anterior temporal pole (ATL).

In sum, there are several cognitive and neural factors that (potentially) play a role in explaining why there is variation between parents in the degree to which they employ gender socialization with their children. The findings are summarized in Figure 1, which visualizes the neural and cognitive factors that were either theoretically or empirically related to parental gender socialization in our synthesis of the literature. In the following paragraphs, these findings are summarized, followed by description of limitations. This section concludes with several recommendations for future research and the social and practical implications of this review.

First, to summarize the cognitive processes, evidence exists that parents’ gender stereotypes and attitudes are implicated in different aspects of gender socialization of children as well as adolescents. There is also some evidence for a link between parents’ gender attributions of the behavior of boys and girls and parents’ differential treatment of boys and girls. For other cognitive factors, such as internal motivation for parenting without gender stereotypes, gender identity, conflict resolution, and intergroup attitudes, theoretical grounding can be provided that these factors might underlie gender socialization. Moreover, gender stereotypes about other domains than toys, gender roles, and academic achievements are likely to play a role in the ways in which parents apply gender socialization. Additional evidence shows that these gender cognitions are implicated in other forms of stereotyped behavior than gender socialization, such as discriminative behavior toward other-gender or other-race individuals, involvement with family roles, or gender-biased voting. Yet, more empirical evidence is necessary to support the association between these cognitive processes and specific gender socialization domains (e.g., role modeling or creating a gendered environment for children).

Second, regarding the neural processes, neural networks associated with attention allocation, salience processing, conflict monitoring, and reward processing, are activated in parents when they are exposed to gendered child stimuli, and this neural processing was associated with the gender socialization they employed with parents’ own children. There is also evidence from several studies in non-parents that brain areas associated with attention allocation and salience processing (amygdala, TPJ), conflict monitoring (ACC), behavior regulation (dl/dmPFC), and linking person-specific memories to faces (ATL) are implicated in people’s stereotypes and stereotyped responses. However, more research in parents with both boys and girls is necessary to further substantiate the link between the above-mentioned neural processes and actual gender socialization practices with parents’ own children.

### Limitations

The current review summarized several cognitive and neural processes that are theoretically or empirically related to parental gender socialization. However, some caveats must be mentioned. First, it is important to note that there is still little research investigating the neural and cognitive processes that may be underlying parental gender socialization. Moreover, for many included studies, the main aim was not to examine the neural or cognitive processes underlying gender socialization and these associations were often part of descriptive or additional analyses.

Furthermore, many studies that have informed the neural network of stereotypes have examined the neural processing of racial stereotypes. However, some precautions must be made before generalizing results from studies on racial stereotypes to gender stereotypes and gender socialization. Racial studies have often examined the neural correlates of race bias under the assumption that people react differently to in-group than to out-group members. However, in-group biases in men and women do not necessarily correlate with their gender expectations (Rudman and Goodwin, 2004). The neural processes implicated in racial stereotypes need to be further evaluated, to see if these processes are also implicated in the context of gender socialization. There is also a general note of caution for interpreting EEG and fMRI studies, because of the often small sample sizes and contradictory findings. Therefore, future research with larger sample sizes is necessary to investigate the neural processes underlying parental gender socialization in the home context.

In addition, the current overview focuses on parents’ gender socialization with their children across childhood and adolescence, but the number of studies that focused on the correlates of parental gender socialization during adolescence was limited. It seems likely that different types of gender socialization (e.g., sexuality, autonomy) are more relevant during teenage years than during early childhood. More research on the processes underlying parents’ gender socialization during adolescence is needed to examine whether additional mechanisms emerge during parental gender socialization with adolescents.

Moreover, the studies described in this paper examined predictors of gender socialization in primarily heterosexual and cisgender parents and toward cisgender children. Even though there is evidence that LGBTQ+ parents are more similar than different than heterosexual parents in their gender socialization practices (Averett, 2016; Bergstrom-Lynch, 2020), it is still important for future research to investigate whether similar neurocognitive processes underlie gender socialization in LGBTQ+ parents and nonbinary or transgender children. For example, the relative importance and strength of association with each neurocognitive process might be different. LGBTQ+ parents might have less strong gender stereotypes through their own gender nonconforming preferences and behaviors and therefore serve as more diverse gender role models for their children (Averett, 2016; Kuvalanka et al., 2018). Similarly, because of their gender nonconforming identity, LGBTQ+ parents might be more motivated to parent without stereotypes, allowing parents to overcome their own gendered beliefs of how a girl or a boy should behave.

Finally, the current overview mainly includes studies with non-Hispanic White US and European families, with the exemption of two studies conducted among Latinx families. However, culture also influences parents’ gender socialization, since it prescribes the gender norms that are ascribed to each gender. For example, Mexican American parents with stronger orientations toward traditional Mexican culture were more likely than parents oriented toward American culture to treat their sons and daughters differently (McHale et al., 2005). The processes presented in the current overview should also be examined in other cultural populations, to examine whether the mechanisms proposed in this study can be generalized toward other non-Western populations. Relatedly, as many other factors interact with gender, such as socioeconomic status, ethnicity, or social class, future research on the processes underlying gender socialization should take a more intersectional approach. Such research could for instance examine differences in the relative importance of each neurocognitive process for parental gender socialization at the intersection of gender and ethnicity, or at the intersection of ethnicity and socioeconomic status.

### Recommendations for future research

Thus far, very few studies have associated gender cognitions and neural processes with gender socialization. Understanding determinants for parent’s engagement in gender socialization is important, as these determinants can be targeted in interventions to reduce traditional gender socialization or foster more gender-neutral socialization (Kok et al., 2016). Therefore, more empirical research is necessary to validate the relevance of the neural and cognitive factors identified in this review for gender socialization across childhood and adolescence. In general, parental gender socialization research could benefit from studies that examine the contributions of several gender cognitions, such as gender identity, gender attributions, and intergroup attitudes on parents’ gender-differentiated parenting with a multi-method approach including observations, self-report questionnaires and/or IATs. Table 1 provides an overview of measures that can be used to assess these cognitive and neural processes in future research.

**Table 1:** Methods to assess neurocognitive processes underlying parental implicit gender socialization.

Methods
*Cognitive processes*	
Gender stereotypes and attitudes	Implicit Association Tests (e.g., Endendijk et al., 2017)
	Self-report questionnaires (e.g., Friedman et al., 2007)
Internal motivation for parenting without gender stereotypes	Self-report questionnaire assessing internal motivation regarding parenting own son(s) and/or daughter(s) (e.g., Endendijk et al., 2019a)
Conflict resolution	Mouse-tracking paradigm (e.g., Hehman et al., 2014a)
Gender attributions	Scenarios, vignettes, pictures (e.g., Morrongiello and Hogg, 2004)
Gender identity	Self-report questionnaire (e.g., Dinella et al., 2014)
Intergroup attitudes	Implicit Association Tasks (e.g., Rudman and Goodwin, 2004)
	Self-reported evaluations of gender ingroup and outgroup (e.g., Rudman and Goodwin, 2004)
*Neural processes*	
	EEG, fMRI, together with:– Passive viewing paradigm (e.g., Endendijk et al., 2019a)– Priming task (e.g., Hehman et al., 2014c)– Implicit Association Task (e.g., Healy et al., 2015)

Studies that focus on the role of internal motivation for parenting without gender stereotypes could additionally investigate the direct and potential moderating role of internal motivation on parents’ gender socialization practices. For example, if parents are aware of the implicit nature in which they steer their sons and daughters into traditional gender-roles, they may be more hesitant to employ these parenting strategies. As a result, parents may be more attentive of their gender socialization practices and increase their motivation to refrain from employing parental gender socialization strategies.

With regard to the neural processes, Mascaro et al.’s (2017) study provided the first evidence of associations between neural responses to stimuli of parents’ own children and differences in play styles with sons and daughters. However, this study examined gender differences in neural responses and play style by comparing fathers of sons with fathers of daughters. Therefore, the authors were unable to directly relate a difference in neural responses to gendered stimuli of sons versus daughters to a difference in gender socialization with sons versus daughters. In order to test such a direct relation, a within-family design is necessary including parents who have both a son and a daughter. Within-family designs are also essential to make sure that differences found in neural and observational responses to boys and girls are not caused by other factors than child gender (McHale et al., 2003; Endendijk et al., 2018).

Lastly, based on the available research, it seems likely that individual differences in the neural processing of stimuli that violate versus confirm gendered expectations are related to individual differences in gender socialization practices. It is therefore recommended that future studies examine whether individual differences in neural responsivity are related to parents’ gender socialization practices with their sons and daughters by combining neuroscientific measures with observational data.

### Social and practical implications

The research findings that were highlighted in this paper have several social and practical implications. First, it stresses the need to examine why some parents are more or less likely to employ gender socialization practices than others. Moreover, several factors that are highlighted in this study might provide useful targets for parenting interventions or psycho-education aimed at increasing gender equality in future generations. Parents’ internal motivation to parent without gender stereotypes might be the most promising factor for intervention as internal motivation to behave non-prejudiced has been found to suppress both the activation of stereotypes as well as the influence of stereotypes on one’s behavior. Similarly, targeting essentialists beliefs about gender in interventions could decrease negative reactions toward (parents of) gender-nonconforming children (Skewes et al., 2018; Sullivan et al., 2018). More gender equal upbringing would decrease the limitations children experience with regard to toy preferences, activities, occupations, and friendship opportunities (Updegraff et al., 1996; Martin et al., 2017; Endendijk and Portengen, 2021).

## Conclusion

To conclude, we have indicated several cognitive and neural factors and processes that could explain why parents differ in the extent to which they employ parental gender socialization. In addition, we provided several suggestions for future research methods that can be used to study these neurocognitive processes and factors. The field particularly needs more research that relates parental cognitive factors, such as internal motivation, conflict resolution, gender identity, and intergroup attitudes, and neural processes, such as behavioral control and reward processing, to different types of gender socialization. This overview of neurocognitive processes associated with parental (implicit) gender socialization, and the predictions that originate from this model, aim to spark and inspire future research in this domain.

## Author contributions

CP and JE contributed to the conceptualization and organization of the manuscript. CP wrote the first drafts. JE and AB provided feedback and supervision during the process. All authors contributed to manuscript revision, read, and approved the submitted version.

## Conflict of interest

The authors declare that the research was conducted in the absence of any commercial or financial relationships that could be construed as a potential conflict of interest.

## Publisher’s note

All claims expressed in this article are solely those of the authors and do not necessarily represent those of their affiliated organizations, or those of the publisher, the editors and the reviewers. Any product that may be evaluated in this article, or claim that may be made by its manufacturer, is not guaranteed or endorsed by the publisher.

## References

[ref1] Abele A. E. (2003). The dynamics of masculine-agentic and feminine-communal traits: findings from a prospective study. J. Pers. Soc. Psychol. 85, 768–776. doi: 10.1037/0022-3514.85.4.768, PMID: 14561129

[ref2] Amodio D. M. (2014). The neuroscience of prejudice and stereotyping. Nat. Rev. Neurosci. 15, 670–682. doi: 10.1038/nrn3800 25186236

[ref3] Amodio D. M. Devine P. G. Harmon-Jones E. (2008). Individual differences in the regulation of intergroup bias: the role of conflict monitoring and neural signals for control. J. Pers. Soc. Psychol. 94, 60–74. doi: 10.1037/0022-3514.94.1.60, PMID: 18179318

[ref4] Amodio D. M. Swencionis J. K. (2018). Proactive control of implicit bias: a theoretical model and implications for behavior change. J. Pers. Soc. Psychol. 115, 255–275. doi: 10.1037/pspi0000128, PMID: 30024242

[ref5] Averett K. H. (2016). The gender buffet: LGBTQ parents resisting Heteronormativity. Gend. Soc. 30, 189–212. doi: 10.1177/0891243215611370

[ref6] Azizian A. Freitas A. L. Parvaz M. A. Squires N. K. (2006). Beware misleading cues: perceptual similarity modulates the N2/P3 complex. Psychophysiology 43, 253–260. doi: 10.1111/j.1469-8986.2006.00409.x, PMID: 16805863

[ref7] Bandura A. (1969). “Social-learning theory of identificatory processes” in Handbook of Socialization Theory and Research (pp. 213–262). ed. Goslin D. A. (Chicago, IL: Rand McNally & Company)

[ref8] Bandura A. Walters R.H. (1977). Social Learning Theory. Englewood Cliffs, NJ: Prentice-Hall.

[ref9] Bartholow B. D. Dickter C. L. (2007). “Social cognitive neuroscience of person perception: a selective review focused on the event-related brain potential” in Social Neuroscience: Integrating Biological and Psychological Explanations of Social Behavior. eds. Harmon-Jones E. Winkielman P. (New York: Guilford Press), 376–400.

[ref10] Bastian B. Haslam N. (2006). Psychological essentialism and stereotype endorsement. J. Exp. Soc. Psychol. 42, 228–235. doi: 10.1016/j.jesp.2005.03.003

[ref11] Bem S. L. (1981). Gender schema theory: a cognitive account of sex typing. Psychol. Rev. 88, 354–364. doi: 10.1037/0033-295X.88.4.354

[ref12] Bem S. L. (1983). Gender schema theory and its implications for child development: raising gender-aschematic children in a gender-schematic society. Signs J. Women Cult. Soc. 8, 598–616. doi: 10.1086/493998

[ref13] Bergstrom-Lynch C. (2020). Free to be you and me, maybe: lesbian, gay, bisexual, and transgender parents doing gender with their children. J. Gend. Stud. 29, 282–294. doi: 10.1080/09589236.2019.1635000

[ref14] Bhanot R. Jovanovic J. (2005). Do parents’ academic gender stereotypes influence whether they intrude on their children’s homework? Sex Roles 52, 597–607. doi: 10.1007/s11199-005-3728-4

[ref15] Blakemore J.E.O. Berenbaum S.A. Liben L.S. (2008). Gender Development. New York, NY: Psychology Press.

[ref16] Bluemke M. Friese M. (2008). Reliability and validity of the single-target IAT (ST-IAT): assessing automatic affect towards multiple attitude objects. Eur. J. Soc. Psychol. 38, 977–997. doi: 10.1002/ejsp.487

[ref17] Bugental D. B. Corpuz R. (2019). “Parental attributions” in Handbook of Parenting: Being and Becoming a Parent, 3rd Edn. ed. Bornstein M. H., vol. 3 (New York, NY: Routledge/Taylor & Francis Group), 722–761.

[ref18] Bugental D. B. Johnston C. (2000). Parental and child cognitions in the context of the family. Annu. Rev. Psychol. 51, 315–344. doi: 10.1146/annurev.psych.51.1.315 10751974

[ref19] Bumpus M. F. Crouter A. C. McHale S. M. (2001). Parental autonomy granting during adolescence: exploring gender differences in context. Dev. Psychol. 37, 163–173. doi: 10.1037/0012-1649.37.2.163, PMID: 11269385

[ref20] Bussey K. Bandura A. (1984). Influence of gender constancy and social power on sex-linked modeling. J. Pers. Soc. Psychol. 47, 1292–1302. doi: 10.1037/0022-3514.47.6.1292, PMID: 6527216

[ref21] Bussey K. Bandura A. (1999). Social cognitive theory of gender development and differentiation. Psychol. Rev. 106, 676–713. doi: 10.1037/0033-295X.106.4.676 10560326

[ref22] Butz D. A. Plant E. A. (2009). Prejudice control and interracial relations: the role of motivation to respond without prejudice. J. Pers. 77, 1311–1342. doi: 10.1111/j.1467-6494.2009.00583.x, PMID: 19686455

[ref23] Canal P. Garnham A. Oakhill J. (2015). Beyond gender stereotypes in language comprehension: self sex-role descriptions affect the brain’s potentials associated with agreement processing. Front. Psychol. 6:1953. doi: 10.3389/fpsyg.2015.01953, PMID: 26779046 PMC4689154

[ref24] Cattaneo Z. Mattavelli G. Platania E. Papagno C. (2011). The role of the prefrontal cortex in controlling gender-stereotypical associations: a TMS investigation. NeuroImage 56, 1839–1846. doi: 10.1016/j.neuroimage.2011.02.037, PMID: 21338690

[ref25] Chaplin T. M. Cole P. M. Zahn-Waxler C. (2005). Parental socialization of emotion expression: gender differences and relations to child adjustment. Emotion 5, 80–88. doi: 10.1037/1528-3542.5.1.80, PMID: 15755221

[ref26] Cloutier J. Gabrieli J. D. O'Young D. Ambady N. (2011). An fMRI study of violations of social expectations: when people are not who we expect them to be. NeuroImage 57, 583–588. doi: 10.1016/j.neuroimage.2011.04.051 21569855

[ref27] Denner J. Laursen B. Dickson D. Hartl A. C. (2016). Latino children’s math confidence: the role of mothers’ gender stereotypes and involvement across the transition to middle school. J. Early Adolesc. 38, 513–529. doi: 10.1177/0272431616675972

[ref28] Derks B. Scheepers D. Ellemers N. (2013). Neuroscience of Prejudice and Intergroup Relations. New York: Psychology Press.

[ref29] Dickter C. Gyurovski I. (2012). The effects of expectancy violations on early attention to race in an impression-formation paradigm. Soc. Neurosci. 7, 240–251. doi: 10.1080/17470919.2011.609906, PMID: 21919565

[ref30] Dinella L. M. Fulcher M. Weisgram E. S. (2014). Sex-typed personality traits and gender identity as predictors of young adults’ career interests. Arch. Sex. Behav. 43, 493–504. doi: 10.1007/s10508-013-0234-6, PMID: 24452631

[ref31] Dittman C. K. Sprajcer M. Turley E. L. (2022). Revisiting gendered parenting of adolescents: understanding its effects on psychosocial development. Curr. Psychol., 1–13. doi: 10.1007/s12144-022-03536-7, PMID: 35967502 PMC9364298

[ref32] Dunham Y. Baron A. S. Banaji M. R. (2016). The development of implicit gender attitudes. Dev. Sci. 19, 781–789. doi: 10.1111/desc.12321 26260250

[ref200] Endendijk J. J. Groeneveld M. G. Bakermans-Kranenburg M. J. Mesman J. (2016). Gender-differentiated parenting revisited: Meta-analysis reveals very few differences in parental control of boys and girls. Plos One, 11, e0159193.. doi: 10.1371/journal.pone.0159193, PMID: 27416099 PMC4945059

[ref33] Endendijk J. J. Groeneveld M. G. Mesman J. (2018). The gendered family process model: an integrative framework of gender in the family. Arch. Sex. Behav. 47, 877–904. doi: 10.1007/s10508-018-1185-8, PMID: 29549542 PMC5891573

[ref34] Endendijk J. J. Groeneveld M. G. van der Pol L. D. van Berkel S. R. Hallers-Haalboom E. T. Bakermans-Kranenburg M. J.. (2017). Gender differences in child aggression: relations with gender-differentiated parenting and parents’ gender-role stereotypes. Child Dev. 88, 299–316. doi: 10.1111/cdev.12589, PMID: 27377595

[ref35] Endendijk J. J. Groeneveld M. G. Van der Pol L. D. Van Berkel S. R. Hallers-Haalboom E. T. Mesman J.. (2014). Boys don’t play with dolls: mothers’ and fathers’ gender talk during picture book reading. Parenting 14, 141–161. doi: 10.1080/15295192.2014.972753

[ref36] Endendijk J. J. Portengen C. M. (2021). Children's views about their future career and family involvement: associations with children's gender schemas and parents' involvement in work and family roles. Front. Psychol. 12:789764. doi: 10.3389/fpsyg.2021.789764, PMID: 35126242 PMC8809201

[ref37] Endendijk J. J. Smit A. K. van Baar A. L. Bos P. A. (2019a). Boys' toys, girls' toys: an fMRI study of mothers' neural responses to children violating gender expectations. Biol. Psychol. 148:107776. doi: 10.1016/j.biopsycho.2019.107776, PMID: 31568818

[ref38] Endendijk J. J. Spencer H. Bos P. A. Derks B. (2019b). Neural processing of gendered information is more robustly associated with mothers’ gendered communication with children than mothers’ implicit and explicit gender stereotypes. Soc. Neurosci. 14, 300–312. doi: 10.1080/17470919.2018.1468357, PMID: 29676664

[ref39] Ex C. T. G. M. Janssens J. M. A. M. (1998). Maternal influences on daughters' gender role attitudes. Sex Roles 38, 171–186. doi: 10.1023/A:1018776931419

[ref40] Feldman R. (2015). The adaptive human parental brain: implications for children's social development. Trends Neurosci. 38, 387–399. doi: 10.1016/j.tins.2015.04.004, PMID: 25956962

[ref41] Fivush R. Brotman M. A. Buckner J. P. Goodman S. H. (2000). Gender differences in parent–child emotion narratives. Sex Roles 42, 233–253. doi: 10.1023/A:1007091207068

[ref42] Friedman C. K. Leaper C. Bigler R. S. (2007). Do mothers' gender-related attitudes or comments predict young children's gender beliefs? Parenting. Sci. Pract. 7, 357–366. doi: 10.1080/15295190701665656

[ref43] Gaunt R. Pinho M. (2018). Do sexist mothers change more diapers? Ambivalent sexism, maternal gatekeeping, and the division of childcare. Sex Roles 79, 176–189. doi: 10.1007/s11199-017-0864-6

[ref44] Gawronski B. Bodenhausen G. V. (2006). Associative and propositional processes in evaluation: an integrative review of implicit and explicit attitude change. Psychol. Bull. 132, 692–731. doi: 10.1037/0033-2909.132.5.692, PMID: 16910748

[ref45] Gawronski B. Bodenhausen G. V. Mitchell C. J. De Houwer J. Lovibond P. F. (2009). Operating principles versus operating conditions in the distinction between associative and propositional processes. Behav. Brain Sci. 32, 207–208. doi: 10.1017/S0140525X09000958

[ref46] Gawronski B. Creighton L. A. (2013). “Dual process theories” in The Oxford Handbook of Social Cognition. ed. Carlston D. E. (New York, NY: Oxford University Press)

[ref47] Gelman S. A. Taylor M. G. Nguyen S. P. (2004). Mother-child conversations about gender: understanding the acquisition of essentialist beliefs. Monogr. Soc. Res. Child Dev. 69, 93–115. doi: 10.1111/j.1540-5834.2004.06901007.x

[ref48] Gilbert S. J. Swencionis J. K. Amodio D. M. (2012). Evaluative vs. trait representation in intergroup social judgments: distinct roles of anterior temporal lobe and prefrontal cortex. Neuropsychologia 50, 3600–3611. doi: 10.1016/j.neuropsychologia.2012.09.002, PMID: 22975194

[ref49] Greenwald A. G. Banaji M. R. Rudman L. A. Farnham S. D. Nosek B. A. Mellott D. S. (2002). A unified theory of implicit attitudes, stereotypes, self-esteem, and self-concept. Psychol. Rev. 109, 3–25. doi: 10.1037/0033-295X.109.1.3, PMID: 11863040

[ref50] Greenwald A. G. Krieger L. H. (2006). Implicit bias: scientific foundations. Calif. Law Rev. 94, 945–967. doi: 10.2307/20439056

[ref51] Greenwald A. G. Pettigrew T. F. (2014). With malice toward none and charity for some: Ingroup favoritism enables discrimination. Am. Psychol. 69, 669–684. doi: 10.1037/a0036056, PMID: 24661244

[ref52] Greenwald A. G. Poehlman T. A. Uhlmann E. L. Banaji M. R. (2009). Understanding and using the implicit association test: III. Meta-analysis of predictive validity. J. Pers. Soc. Psychol. 97, 17–41. doi: 10.1037/a0015575, PMID: 19586237

[ref53] Halpern H. P. Perry-Jenkins M. (2016). Parents’ gender ideology and gendered behavior as predictors of children’s gender-role attitudes: a longitudinal exploration. Sex Roles 74, 527–542. doi: 10.1007/s11199-015-0539-0, PMID: 27445431 PMC4945126

[ref54] He Y. Johnson M. K. Dovidio J. F. McCarthy G. (2009). The relation between race-related implicit associations and scalp-recorded neural activity evoked by faces from different races. Soc. Neurosci. 4, 426–442. doi: 10.1080/17470910902949184, PMID: 19562628 PMC2755624

[ref55] Healy G. F. Boran L. Smeaton A. F. (2015). Neural patterns of the implicit association test. Front. Hum. Neurosci. 9:605. doi: 10.3389/fnhum.2015.00605, PMID: 26635570 PMC4656831

[ref56] Hehman E. Carpinella C. M. Johnson K. L. Leitner J. B. Freeman J. B. (2014a). Early processing of gendered facial cues predicts the electoral success of female politicians. Soc. Psychol. Personal. Sci. 5, 815–824. doi: 10.1177/1948550614534701

[ref57] Hehman E. Ingbretsen Z. A. Freeman J. B. (2014b). The neural basis of stereotypic impact on multiple social categorization. NeuroImage 101, 704–711. doi: 10.1016/j.neuroimage.2014.07.056, PMID: 25094016

[ref58] Hehman E. Volpert H. I. Simons R. F. (2014c). The N400 as an index of racial stereotype accessibility. Soc. Cogn. Affect. Neurosci. 9, 544–552. doi: 10.1093/scan/nst018, PMID: 23386742 PMC3989137

[ref59] Henslin J.M. (1981). Down to Earth Sociology:: Introductory Readings. New York: Simon and Schuster.

[ref60] Huffmeijer R. Bakermans-Kranenburg M. J. Alink L. R. Van IJzendoorn M. H. (2014). Reliability of event-related potentials: the influence of number of trials and electrodes. Hormones Behav. 130, 13–22. doi: 10.1016/j.yhbeh.2012.11.008, PMID: 24642000

[ref61] Kane E. W. (2006). “No way my boys are going to be like that!” parents’ responses to children’s gender nonconformity. Gend. Soc. 20, 149–176. doi: 10.1177/0891243205284276

[ref62] Knutson K. M. Mah L. Manly C. F. Grafman J. (2007). Neural correlates of automatic beliefs about gender and race. Hum. Brain Mapp. 28, 915–930. doi: 10.1002/hbm.20320, PMID: 17133388 PMC6871386

[ref63] Kok G. Gottlieb N. H. Peters G.-J. Y. Mullen P. D. Parcel G. S. Ruiter R. A. C.. (2016). A taxonomy of behaviour change methods: an intervention mapping approach. Health Psychol. Rev. 10, 297–312. doi: 10.1080/17437199.2015.1077155, PMID: 26262912 PMC4975080

[ref64] Kollmayer M. Schober B. Spiel C. (2018). Gender stereotypes in education: development, consequences, and interventions. Eur. J. Dev. Psychol. 15, 361–377. doi: 10.1080/17405629.2016.1193483

[ref65] Kuvalanka K. A. Allen S. H. Munroe C. Goldberg A. E. Weiner J. L. (2018). The experiences of sexual minority mothers with trans* children. Fam. Relat. 67, 70–87. doi: 10.1111/fare.12226

[ref66] Li T. Cardenas-Iniguez C. Correll J. Cloutier J. (2016). The impact of motivation on race-based impression formation. NeuroImage 124, 1–7. doi: 10.1016/j.neuroimage.2015.08.035, PMID: 26302673

[ref67] Lilford R. J. Richardson A. Stevens A. Fitzpatrick R. Edwards S. Rock F.. (2001). Issues in methodological research: perspectives from researchers and commissioners. Health Technol. Assess. 5, 1, –57. doi: 10.3310/hta5080 11368832

[ref68] Martin C. L. (1995). Stereotypes about children with traditional and nontraditional gender roles. Sex Roles 33, 727–751. doi: 10.1007/bf01544776

[ref69] Martin C. L. Fabes R. A. Hanish L. D. Gaertner B. Miller C. F. Foster S.. (2017). “Using an intergroup contact approach to improve gender relationships” in The Wiley Handbook of Group Processes in Children and Adolescents. eds. Rutland A. Nesdale D. Brown C. S. (New York: Wiley), 435–454.

[ref70] Martin C. L. Halverson C. F. (1981). A schematic processing model of sex typing and stereotyping in children. Child Dev. 52:1119. doi: 10.2307/1129498

[ref71] Martin J. L. Ross H. S. (2005). Sibling aggression: sex differences and parents’ reactions. Int. J. Behav. Dev. 29, 129–138. doi: 10.1080/01650250444000469

[ref72] Mascaro J. S. Rentscher K. E. Hackett P. D. Mehl M. R. Rilling J. K. (2017). Child gender influences paternal behavior, language, and brain function. Behav. Neurosci. 131, 262–273. doi: 10.1037/bne0000199, PMID: 28541079 PMC5481199

[ref73] Maupin A. N. Hayes N. J. Mayes L. C. Rutherford H. J. V. (2015). The application of electroencephalography to investigate the neural bases of parenting: a review. Parenting 15, 9–23. doi: 10.1080/15295192.2015.992735, PMID: 26120286 PMC4477836

[ref74] McHale S. M. Crouter A. C. Tucker C. J. (1999). Family context and gender role socialization in middle childhood: comparing girls to boys and sisters to brothers. Child Dev. 70, 990–1004. doi: 10.1111/1467-8624.00072, PMID: 10446731

[ref75] McHale S. M. Crouter A. C. Whiteman S. D. (2003). The family contexts of gender development in childhood and adolescence. Soc. Dev. 12, 125–148. doi: 10.1111/1467-9507.00225

[ref76] McHale S. M. Updegraff K. A. Shanahan L. Crouter A. C. Killoren S. E. (2005). Siblings’ differential treatment in Mexican American families. J. Marriage Fam. 67, 1259–1274. doi: 10.1111/j.1741-3737.2005.00215.x, PMID: 18414595 PMC2293294

[ref77] Mesman J. Groeneveld M. G. (2018). Gendered parenting in early childhood: subtle but unmistakable if you know where to look. Child Dev. Perspect. 12, 22–27. doi: 10.1111/cdep.12250

[ref78] Meyer M. Gelman S. A. (2016). Gender essentialism in children and parents: implications for the development of gender stereotyping and gender-typed preferences. Sex Roles 75, 409–421. doi: 10.1007/s11199-016-0646-6

[ref79] Mitchell J. P. (2008). Contributions of functional neuroimaging to the study of social cognition. Curr. Dir. Psychol. Sci. 17, 142–146. doi: 10.1111/j.1467-8721.2008.00564.x

[ref80] Morawska A. (2020). The effects of gendered parenting on child development outcomes: a systematic review. Clin. Child. Fam. Psychol. Rev. 23, 553–576. doi: 10.1007/s10567-020-00321-5, PMID: 32681376

[ref81] Morrongiello B. A. Dawber T. (2000). Mothers' responses to sons and daughters engaging in injury-risk behaviors on a playground: implications for sex differences in injury rates. J. Exp. Child Psychol. 76, 89–103. doi: 10.1006/jecp.2000.2572, PMID: 10788304

[ref82] Morrongiello B. A. Hogg K. (2004). Mothers' reactions to children misbehaving in ways that can lead to injury: implications for gender differences in children's risk taking and injuries. Sex Roles 50, 103–118. doi: 10.1023/B:SERS.0000011076.43831.a6

[ref83] Morrongiello B. A. Klemencic N. Corbett M. (2008). Interactions between child behavior patterns and parent supervision: implications for children’s risk of unintentional injury. Child Dev. 79, 627–638. doi: 10.1111/j.1467-8624.2008.01147.x, PMID: 18489417

[ref84] Morrongiello B. A. Rennie H. (1998). Why do boys engage in more risk taking than girls? The role of attributions, beliefs, and risk appraisals. J. Pediatr. Psychol. 23, 33–43. doi: 10.1093/jpepsy/23.1.33, PMID: 9564127

[ref85] Morrongiello B. A. Zdzieborski D. Normand J. (2010). Understanding gender differences in children's risk taking and injury: a comparison of mothers' and fathers' reactions to sons and daughters misbehaving in ways that lead to injury. J. Appl. Dev. Psychol. 31, 322–329. doi: 10.1016/j.appdev.2010.05.004

[ref86] Olson I. R. McCoy D. Klobusicky E. Ross L. A. (2013). Social cognition and the anterior temporal lobes: a review and theoretical framework. Soc. Cogn. Affect. Neurosci. 8, 123–133. doi: 10.1093/scan/nss119, PMID: 23051902 PMC3575728

[ref87] Parke R. D. (2017). Family psychology: past and future reflections on the field. J. Fam. Psychol. 31, 257–260. doi: 10.1037/fam0000318, PMID: 28368174

[ref88] Pinho M. Gaunt R. (2021). Biological essentialism, gender ideologies, and the division of housework and childcare: comparing male carer/female breadwinner and traditional families. J. Soc. Psychol. 1-17, 1–17. doi: 10.1080/00224545.2021.1983508, PMID: 34632955

[ref89] Plant E. A. Devine P. G. (1998). Internal and external motivation to respond without prejudice. J. Pers. Soc. Psychol. 75, 811–832. doi: 10.1037/0022-3514.75.3.811 16055643

[ref90] Polich J. (2007). Updating P300: an integrative theory of P3a and P3b. Clin. Neurophysiol. 118, 2128–2148. doi: 10.1016/j.clinph.2007.04.019, PMID: 17573239 PMC2715154

[ref91] Pruden S. M. Levine S. C. (2017). Parents’ spatial language mediates a sex difference in preschoolers’ spatial-language use. Psychol. Sci. 28, 1583–1596. doi: 10.1177/0956797617711968, PMID: 28880726 PMC5673527

[ref92] Quadflieg S. Flannigan N. Waiter G. D. Rossion B. Wig G. S. Turk D. J.. (2011). Stereotype-based modulation of person perception. NeuroImage 57, 549–557. doi: 10.1016/j.neuroimage.2011.05.004, PMID: 21586332

[ref93] Raffaelli M. Ontai L. L. (2004). Gender socialization in latino/a families: results from two retrospective studies. Sex Roles 50, 287–299. doi: 10.1023/B:SERS.0000018886.58945.06

[ref94] Reyna C. (2000). Lazy, dumb, or industrious: when stereotypes convey attribution information in the classroom. Educ. Psychol. Rev. 12, 85–110. doi: 10.1023/A:1009037101170

[ref95] Rodríguez-Gómez P. Romero-Ferreiro V. Pozo M. A. Hinojosa J. A. Moreno E. M. (2020). Facing stereotypes: ERP responses to male and female faces after gender-stereotyped statements. Soc. Cogn. Affect. Neurosci. 15, 928–940. doi: 10.1093/scan/nsaa117, PMID: 32901810 PMC7647374

[ref96] Rolls E. T. Cheng W. Feng J. (2020). The orbitofrontal cortex: reward, emotion and depression. Brain Commun. 2:fcaa196. doi: 10.1093/braincomms/fcaa196, PMID: 33364600 PMC7749795

[ref97] Rudman L. A. Goodwin S. A. (2004). Gender differences in automatic in-group bias: why do women like women more than men like men? J. Pers. Soc. Psychol. 87, 494–509. doi: 10.1037/0022-3514.87.4.494, PMID: 15491274

[ref98] Rudman L. A. Greenwald A. G. Mellott D. S. Schwartz J. L. (1999). Measuring the automatic components of prejudice: flexibility and generality of the implicit association test. Soc. Cogn. 17, 437–465. doi: 10.1521/soco.1999.17.4.437

[ref99] Sandberg D. E. Ehrhardt A. A. Ince S. E. Meyer-Bahlburg H. F. (1991). Gender differences in children's and adolescents' career aspirations: a follow-up study. J. Adolesc. Res. 6, 371–386. doi: 10.1177/074355489163007

[ref100] Sandnabba N. K. Ahlberg C. (1999). Parents' attitudes and expectations about children's cross-gender behavior. Sex Roles 40, 249–263. doi: 10.1023/A:1018851005631

[ref101] Santos A. Mier D. Kirsch P. Meyer-Lindenberg A. (2011). Evidence for a general face salience signal in human amygdala. NeuroImage 54, 3111–3116. doi: 10.1016/j.neuroimage.2010.11.024, PMID: 21081170

[ref102] Schroeder K. M. Liben L. S. (2021). Felt pressure to conform to cultural gender roles: correlates and consequences. Sex Roles 84, 125–138. doi: 10.1007/s11199-020-01155-9

[ref103] Skewes L. Fine C. Haslam N. (2018). Beyond Mars and Venus: the role of gender essentialism in support for gender inequality and backlash. PLoS One 13:e0200921. doi: 10.1371/journal.pone.0200921, PMID: 30040839 PMC6057632

[ref104] Smetana J. G. (1989). Toddlers' social interactions in the context of moral and conventional transgressions in the home. Dev. Psychol. 25, 499–508. doi: 10.1037/0012-1649.25.4.499

[ref105] Stanley D. Phelps E. Banaji M. (2008). The neural basis of implicit attitudes. Curr. Dir. Psychol. Sci. 17, 164–170. doi: 10.1111/j.1467-8721.2008.00568.x

[ref106] Steensma T. D. Kreukels B. P. de Vries A. L. Cohen-Kettenis P. T. (2013). Gender identity development in adolescence. Hormones Behav. 64, 288–297. doi: 10.1016/j.yhbeh.2013.02.020 23998673

[ref107] Stillman P. E. Shen X. Ferguson M. J. (2018). How mouse-tracking can advance social cognitive theory. Trends Cogn. Sci. 22, 531–543. doi: 10.1016/j.tics.2018.03.012, PMID: 29731415

[ref108] Sullivan J. Moss-Racusin C. Lopez M. Williams K. (2018). Backlash against gender stereotype-violating preschool children. PLoS One 13:e0195503. doi: 10.1371/journal.pone.0195503, PMID: 29630651 PMC5890994

[ref109] Tajfel H. Turner J. (1986). “The social identity theory of intergroup behavior” in Psychology of Intergroup Relations. eds. Worchel S. Austing W. G. (Chicago, IL: Nelson-Hall)

[ref110] Taylor M. C. Hall J. A. (1982). Psychological androgyny: theories, methods, and conclusions. Psychol. Bull. 92, 347–366. doi: 10.1037/0033-2909.92.2.347

[ref111] Tenenbaum H. R. Leaper C. (2002). Are parents' gender schemas related to their children's gender-related cognitions? A meta-analysis. Dev. Psychol. 38, 615–630. doi: 10.1037/0012-1649.38.4.615, PMID: 12090490

[ref112] Twomey D. M. Murphy P. R. Kelly S. P. O'Connell R. G. (2015). The classic P300 encodes a build-to-threshold decision variable. Eur. J. Neurosci. 42, 1636–1643. doi: 10.1111/ejn.12936, PMID: 25925534

[ref113] Updegraff K. A. McHale S. M. Crouter A. C. (1996). Gender roles in marriage: what do they mean for girls' and boys' school achievement? J. Youth Adolesc. 25, 73–88. doi: 10.1007/BF01537381

[ref114] van der Pol L. D. Groeneveld M. G. van Berkel S. R. Endendijk J. J. Hallers-Haalboom E. T. Bakermans-Kranenburg M. J.. (2015). Fathers’ and mothers’ emotion talk with their girls and boys from toddlerhood to preschool age. Emotion 15, 854–864. doi: 10.1037/emo0000085, PMID: 26168009

[ref115] Weisgram E. S. Bruun S. T. (2018). Predictors of gender-typed toy purchases by prospective parents and mothers: the roles of childhood experiences and gender attitudes. Sex Roles 79, 342–357. doi: 10.1007/s11199-018-0928-2

[ref116] Wood W. Eagly A. H. (2015). Two traditions of research on gender identity. Sex Roles 73, 461–473. doi: 10.1007/s11199-015-0480-2

[ref117] Xiao F. Zheng Z. Wang Y. Cui J. Chen Y. (2015). Conflict monitoring and stimulus categorization processes involved in the prosocial attitude implicit association test: evidence from event-related potentials. Soc. Neurosci. 10, 1–10. doi: 10.1080/17470919.2014.1003598, PMID: 25623088

